# Control of *Propionibacterium acnes* by natural antimicrobial substances: Role of the bacteriocin AS-48 and lysozyme

**DOI:** 10.1038/s41598-018-29580-7

**Published:** 2018-08-06

**Authors:** Rubén Cebrián, Sergio Arévalo, Susana Rubiño, Salvador Arias-Santiago, María Dolores Rojo, Manuel Montalbán-López, Manuel Martínez-Bueno, Eva Valdivia, Mercedes Maqueda

**Affiliations:** 1 Microbiology Department, Faculty of Sciences, C/Fuentenueva s/n, University of Granada, 18071 Granada, Spain; 2 Dermatology Department, Virgen de las Nieves Hospital, Carretera de Jaén, s/n, 18013 Granada, Spain; 3 Microbiology Service, Virgen de las Nieves Hospital, Avenida de las Fuerzas Armadas, 2, 18014 Granada, Spain; 4 Present Address: Department of Molecular Genetics, Faculty of Science and Engineering, Nijenborgh 7, 9747 AG, University of Groningen, Groningen, The Netherlands

## Abstract

We report the high susceptibility of several clinical isolates of *Propionibacterium acnes* from different sources (skin, bone, wound exudates, abscess or blood contamination) to the head-to-tail cyclized bacteriocin AS-48. This peptide is a feasible candidate for further pharmacological development against this bacterium, due to its physicochemical and biological characteristics, even when it is growing in a biofilm. Thus, the treatment of pre-formed biofilms with AS-48 resulted in a dose- and time-dependent disruption of the biofilm architecture beside the decrease of bacterial viability. Furthermore, we demonstrated the potential of lysozyme to bolster the inhibitory activity of AS-48 against *P*. *acnes*, rendering high reductions in the MIC values, even in matrix-growing cultures, according to the results obtained using a range of microscopy and bioassay techniques. The improvement of the activity of AS-48 through its co-formulation with lysozyme may be considered an alternative in the control of *P*. *acnes*, especially after proving the absence of cytotoxicity demonstrated by these natural compounds on relevant human skin cell lines. In summary, this study supports that compositions comprising the bacteriocin AS-48 plus lysozyme must be considered as promising candidates for topical applications with medical and pharmaceutical purposes against dermatological diseases such as acne vulgaris.

## Introduction

*Propionibacterium acnes* is a pleomorphic rod belonging to the Phylum *Actinobacteria* that forms part of the normal microbiota of the skin, and also of the oral cavity, and gastrointestinal and genitourinary tracts. It is a member of the commensal skin microbiota of virtually every human, and it is by far the most prevalent in pilosebaceous follicles. Its association with acne vulgaris has been established in spite of its role being still somewhat controversial^1–6^. Acne occurs in areas with higher densities of pilosebaceous units, as a multifactorial response (hormonal, microbiological, and immunological mechanisms)^7^. Currently *P*. *acnes* is also considered as an opportunistic pathogen in infections linked to surgical procedures, foreign bodies, septicemia, and in implant-associated infections (prosthetic joints, breast fibrosis, cardiovascular device-related infections or spinal osteomyelitis)^4,6,8,9^.

Antimicrobial drug resistance is a growing risk to global public health. The widespread use of antibiotics has been associated with the increase in the occurrence of resistant organisms. Many causes are involved in the emergence of resistances (prolonged administration, poor compliance, subdosing, or monotherapy treatment). So, the discovery and development of novel therapeutic drugs with new targets and unique mechanisms of action against drug-resistant pathogens are urgent. One promising approach currently under consideration are the broad-spectrum antimicrobial peptides (AMPs) produced by most living organisms as components of their natural defence against the invading pathogens^10,11^. These are molecules with potent antimicrobial activity and new mechanisms of action, primarily based on their amphiphilic nature and their ability to selectively disrupt microbial membranes, being at the present promising candidates for commercial and clinical uses^12^. The notable expansion in the peptide-based drug discovery field over the past 10 years^13^ encouraged us to test bacteriocins, that are a type of AMPs of ribosomal synthesis secreted by bacteria that inhibit the growth of closely related species (narrow spectrum) or across genera (broad spectrum). The bacteriocins show low toxicity and are generally extremely potent compared with most of their eukaryotic counterparts and for these reason these peptides are biotechnologically relevant in therapy, agriculture and food preservation^14–16^. For instance, nisin is marketed in different countries under the denomination E-234 and has been used in the food industry for more than 50 years. Similarly, a commercial non-purified preparation of pediocin (Alta TM 2341) is available in food biopreservation^17^. Bacteriocinogenic strains can also be applied in this field (e.g. *Carnobacterium maltaromaticum* producing carnocyclin is approved for use)^18^. Moreover, the therapeutic potential of bacteriocins in local and systemic bacterial infections is currently under study, underlining their importance as a viable alternative or as an addition to currently used antibiotics considering the alarming increase of antibiotic resistances^19^. In fact, many bacteriocins fulfill most criteria that encourage further study to market them as clinically relevant compounds, such as low MIC values, low immunogenicity and toxicity, and low tendency to induce resistance^11,12,20,21^. There are some reports about the ability of bacteriocins to inhibit *P*. *acnes*, reducing the inflammatory lesions caused by this bacterium, such as those produced by *Lactococcus s*p. HY 449^22^, *Streptococcus*^23^, *Enterococcus faecalis* SL-5^24,25^ or *Lactobacillus plantarum*^25^.

AS-48 is a 70-residues, gene-encoded, alpha-helical, circular, cationic bacteriocin produced by different *Enterococcus* species. Its potential use as an antimicrobial agent due to its bactericidal action on many Gram-positive and some Gram-negative bacteria^26,27^ and also as an anti-trypanosomide agent^28^ has been reported. As with other bacteriocins from LABs, AS-48 has been characterized and assayed to facilitate its application as a food additive^29^, but its clinical potential is largely unexplored. The most distinctive structural feature of this bacteriocin is, unquestionably, its circular structure, which contributes to the stability of the native form, because of the reduction in conformational entropy^30–32^.

The aim of this work is to examine the effectiveness of AS-48 alone and in combination with lysozyme, an antimicrobial enzyme widely distributed in various biological fuids and tissues, against *P*. *acnes*. Lysozyme forms part of the innate immune system to treat local site-specific infections produced by *P*. *acnes*. The combination of AS-48 and lysozyme was effective against planktonic cells as well as biofilms of *P*. *acnes*, as it has been proved using a range of microscopy and bioassay techniques. The absence of citotoxycity of this combination on cellular lines opens novel prospects for developing topical treatments for the control of this bacterium, both in the skin and mucous membranes, as well as in prostheses avoiding the formation of biofilms.

## Results

### AS-48 is active against clinical *P*. *acnes* isolates. MIC of AS-48 alone and combined with lysozyme

Several clinical isolates of *P*. *acnes* (n = 20) from different sources (Table 1) including three antibiotic-resistant isolates, P3, P11 and P12 previously characterized^33^, were examined for susceptibility to the bacteriocin AS-48. The MIC was determined by the broth microdilution method according to the Clinical and Laboratory Standard Institute (CLSI) guidelines for anaerobic bacteria, although using DRCM broth to promote the growth. As expected, the MIC of AS-48 was strain-dependent regardless the origin, ranging from the most susceptible strains, with values lower than 1.00 µg/mL (P0, P4, P11, P12, P13 from acne; P1 isolate from a wound; and P15, P22 and P27 isolated as contaminants), between 1.00 and 2.00 µg/mL (P3 and P24 from acne; P10 and P25 from wound exudates; and P6, P17, P18, P20 and P26 as contaminants) and finally two strain with AS-48 MICs of 2.50 µg/mL (P7 and P19 as contaminants) (Table 1).

**Table 1:** Minimal inhibitory concentration of AS-48 alone and in combination with lysozyme assayed by the microdilution method.

Isolate	AS-48 (µg/mL)	AS-48 (µg/mL) + Lysozyme (0.40 mg/mL)	Isolate	AS-48 (µg/mL)	AS-48 (µg/mL) + Lysozyme (0.40 mg/mL)
**Acne**			**Other sources**		
P0	0.62	0.052**	P6	1.25	0.86/0.15
P3	1.25	0.26	P7	2.50	—
P4	0.62	—	P15	0.94	0.625
P11	0.62	0.47	P17	1.25	0.104**
P12	0.75	0.019**	P18	1.25	0.93
P13	0.94	0.39	P19	2.50	0.62
P24	1.87	1.87	P20	1.25	0.62
**Wound exudates**			P22	0.62	0.019**
P1	0.94	0.286	P26	1.25	0.62
P10	1.87	0.62	P27	0.92	0.065**
P25	1.25	0.62			

Non-cooperative effect is shown underlined. **Represents the most significant MIC reductions (more than 10x). The results are representative of three or more independent experiments.

We assayed the dose–response effect of AS-48 combined with lysozyme and, to evaluate its effect on the AS-48 activity, we repeated the broth microdilution method, using mixtures of both compounds. In general, the activity of AS-48 was enhanced by lysozyme, even when this enzyme was used at very low concentrations (0.40 mg/mL). In the majority of the cases, the MIC of AS-48 plus lysozyme was lower than that of AS-48 alone, confirming previous results protected by a Spanish patent about the effectiveness of AS-48 and lysozyme against acne and other skin bacterial infections^34^. In spite of the low concentration of lysozyme assayed (0.40 mg/mL), this combination was active against the isolates, with reduced MIC values in 19/20 cases (the exception being the isolate P24), including the erythromycin- (P3) and erythromycin-clindamycin-resistant (P12) strains, where the MIC of AS-48 decreased from 1.25 and 0.75 µg/mL, respectively, to values lower than 0.26 and 0.019 µg/mL with significant differences at statistical level (*p* = 0.000) (results not shown).

### *P*. *acnes* biofilm composition

We have confirmed that several isolates growing in wells of conical-bottoned microtiter plates were able to develop biofilms, although they tended to become dislodged during manipulation^35^. In order to determine the net composition of the biofilms, we carried out biofilm stability assays in the presence of carbohydrate-, protein-, and DNA-dispersal agents^36^. According to the response to these dispersal agents, we can conclude that the biofilms were composed predominantly of proteins and in some cases of e-DNA (results not shown) as it has been published for this bacterium^37^.

### Electron microscopy of *P*. *acnes* P27 biofilms treated with AS-48

The appearance of the matrix of the P27 cells growing in liquid medium was visualized by electronic microscopy using both, SEM and TEM. We conducted several assays to assess the effect of three differentAS-48 concentrations added at two different times. Addition at T_0_ was carried out to interfere with the ability of the cells to form the adherent matrix, and at T_24_, to examine the ability of AS-48 to inhibit cells growing in an existing matrix. For this, the P27 strain was grown in liquid culture with or without a small glass (10 × 10 mm) inside, to be treated with increased AS-48 concentrations (0.10, 1.00 and 10.00 µg/mL) at different times (T_0_ and T_24_) and re-incubated for 48 h. A control without AS-48 was also carried out.

**Figure 1:**
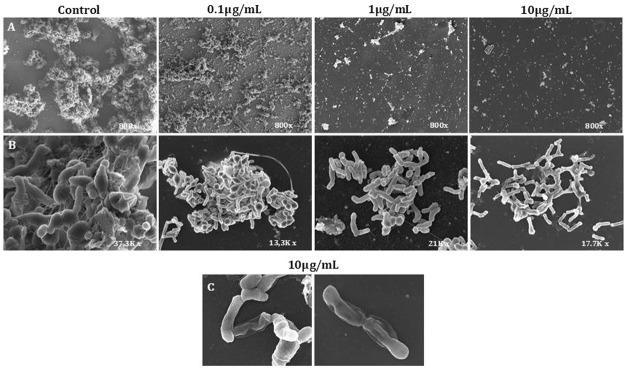
Scanning electron micrographs of the P27 cells growing in a biofilm. (**A**) Massive numbers of bacilli partially occluded by dehydrated material (untreated control) and progressive reduction of cell proliferation by the addition of 0.10, 1.00 and 10.00 µg/mL of AS-48 at T_0_. (**B**) Morphology of the P27 cells added of different AS-48 concentrations at T_0_. (**C**) Membrane-surface retraction on cells treated with high concentrations of AS-48.

**Figure 2:**
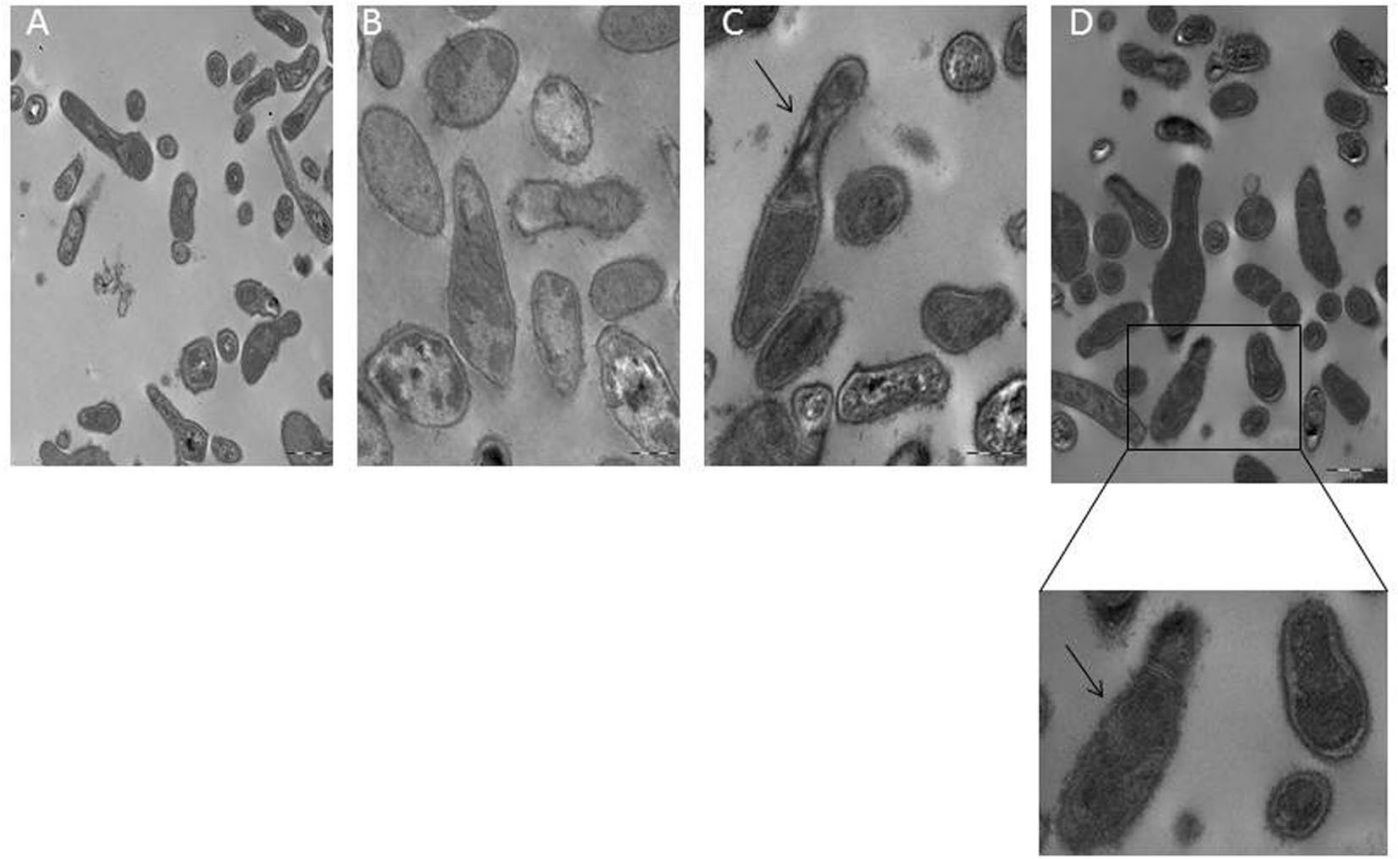
Transmission electron micrographs (TEM) of the P27 cells growing alone (**A**) and in the presence of 0.10 (**B**), 1.00 (**C**) and 10.00 µg/mL (**D**) of AS-48 at T_0_. The arrows shown the membrane-surface retraction on cells treated with high concentrations of AS-48.

SEM images of untreated controls revealed the existence of large cell aggregates recognizable as a biofilm, which biomass was visibly reduced in the presence of AS-48 in a dose- and time-dependent manner (Fig. 1A). Remarkably, lysis was not observed at any of the AS-48 concentrations assayed, either at T_0_ or T_24_, as it has already been described in other bacteria belonging to the Phylum *Actinobacteria* exposed to AS-48^26,38^. Likewise, the treated cells showed no significant morphological alterations as compared with the control, although at the higest AS-48 concentration used (10.00 µg/mL), the cells lost the turgor and the typical pleomorphism was reduced (Fig. 1B). Our results with scanning electron microscropy visualization appear to show a membrane-surface retractions on the cells, due to a loss of volume of the cellular content, while maintaining the integrity of the cell wall where a large gap could be appreciated (Fig. 1C). The membrane-surface retractions on the cells at the highest AS-48 concentration used have been confirmed in TEM microphotografies (Fig. 2C,D). It is likely that the mode of action of individual peptides may vary according to the particular bacterial target cell, the concentration at which they are assayed, and the physical properties of the interacting membrane^39^.

**Figure 3:**
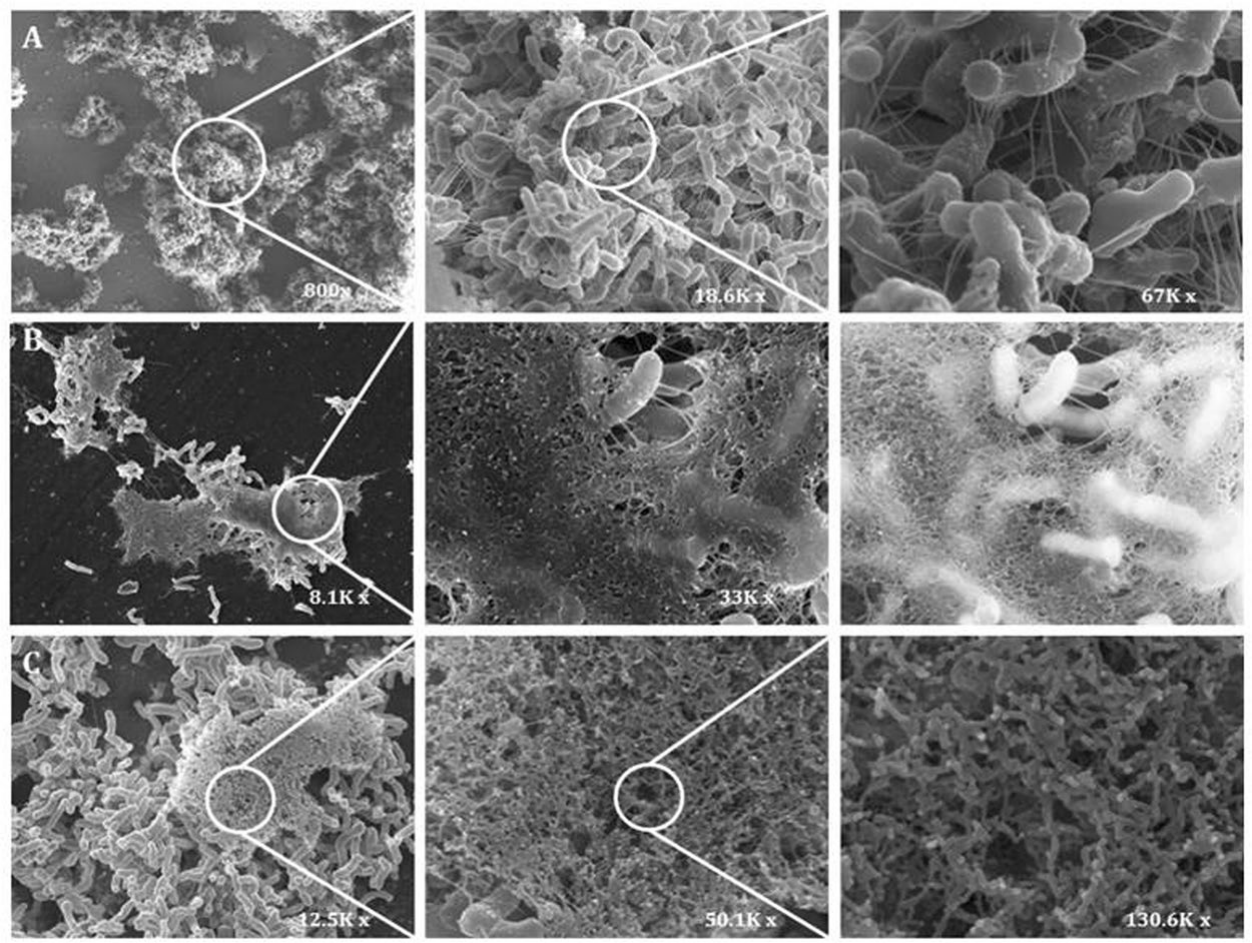
Effect of AS-48 on P27 cells growing in a biofilm visualized by SEM at increasing magnifications at T_24_. (**A**) Bacilli are embedded in fimbriae-like structures (control). (**B**) Networks of fibrils surrounding and entangling the bacilli after AS-48 addition (1.00 µg/mL). (**C)** Details of the networks with a progressive higher magnification.

When AS-48 was added to 24 h-old biofilms, bacilli in the control were visualized embedded in an exopolymeric matrix (fimbriae-like structures) (Fig. 3A). Nevertheless, regarding the effect of AS-48 on cells, several networks of fibrils surrounding the bacterial surface and entangling the bacteria, absent in the control, were discernible particularly at AS-48 MIC value (1.00 µg/mL, considering the MIC of planktonic cells) (Fig. 3B). Details of the networks with a progressive higher magnification could be seen in Fig. 3C. We tested the viability of treated cells within the biofilm after the challenge with AS-48. In a preliminary assay on the proliferative capacity of P27 cells after AS-48 treatment, we confirmed that after addition of 1.00 µg/mL of AS-48 or higher, it was not possible to recover bacterial growth on bacteriocin-free media^40^.

### Flow cytometry analysis of cell membrane damage

The dynamic of live/dead of the P0 cells growing in a biofilm in the presence of AS-48 (16.00 to 0.065 µg/mL), either alone or added of lysozyme (4.00 mg/mL), have been studied by flow cytometry, using a combination of fluorescent markers (acridine orange and propidium iodide)^41^.

**Figure 4:**
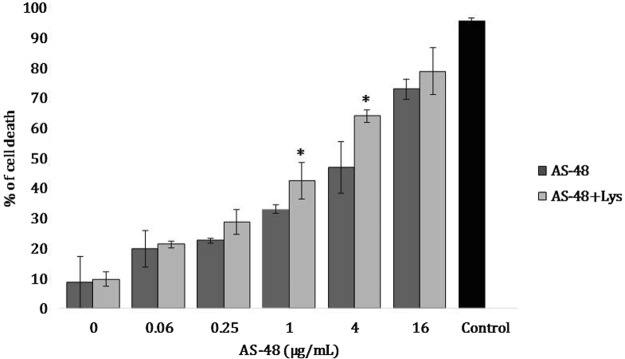
Flow cytometry analysis of *P*. *acnes *P0 strain growing in biofilms and exposed to several concentrations of AS-48 and AS-48 plus lysozyme, stained with acridine orange and propidium iodide^41^. Percentage of dead cells after the tratments, according to the flow cytometry analysis in relation to control of death (80 °C, 20 min). *Significant differences according to Anova one-way Posthoc LSD (*p* < 0.05).

In the control, P0 cells growing in anaerobiosis during 24 h to allow the formation of a biofilm, the bacterial-cell population was mostly alive with no bacterial-cell death detected. Conversely, AS-48 addition produced a progressive shift in the flow-cytometer profile, where an increase of the proportion of dead cells could be observed (Fig. 4). These results are not comparable with those of MIC, given the higher cell density used in this assay. The decrease of the P0 survivors was a function of the concentrations used, confirming the activity of AS-48 on the cell membrane permeability, and the cooperative effect of the lysozyme (Fig. 4).

The size and complexity of the populations (untreated control and cells treated with 16.00 µg/mL AS-48) were also indicative of the absence of lysis (the size and morphology of the majority of the cells was similar in treated and nontreated cells) (results not shown). The results are consistent with flow cytometry experiments in this bacterium using a nucleic acid dye (propidium iodide). Progressive reduction in the percentage of survivors in the presence of AS-48 (with merely 0.06 µg/mL of AS-48, there was already a rate of 20% dead cells) could be clearly monitored. In all cases, the addition of lysozyme increased the death cell rate. Thus, we confirmed a cooperative effect of lysozyme on the bactericidal activity of AS-48, even on biofilm-embedded cells.

### Killing kinetics of AS-48 against *P*. *acnes*

**Figure 5:**
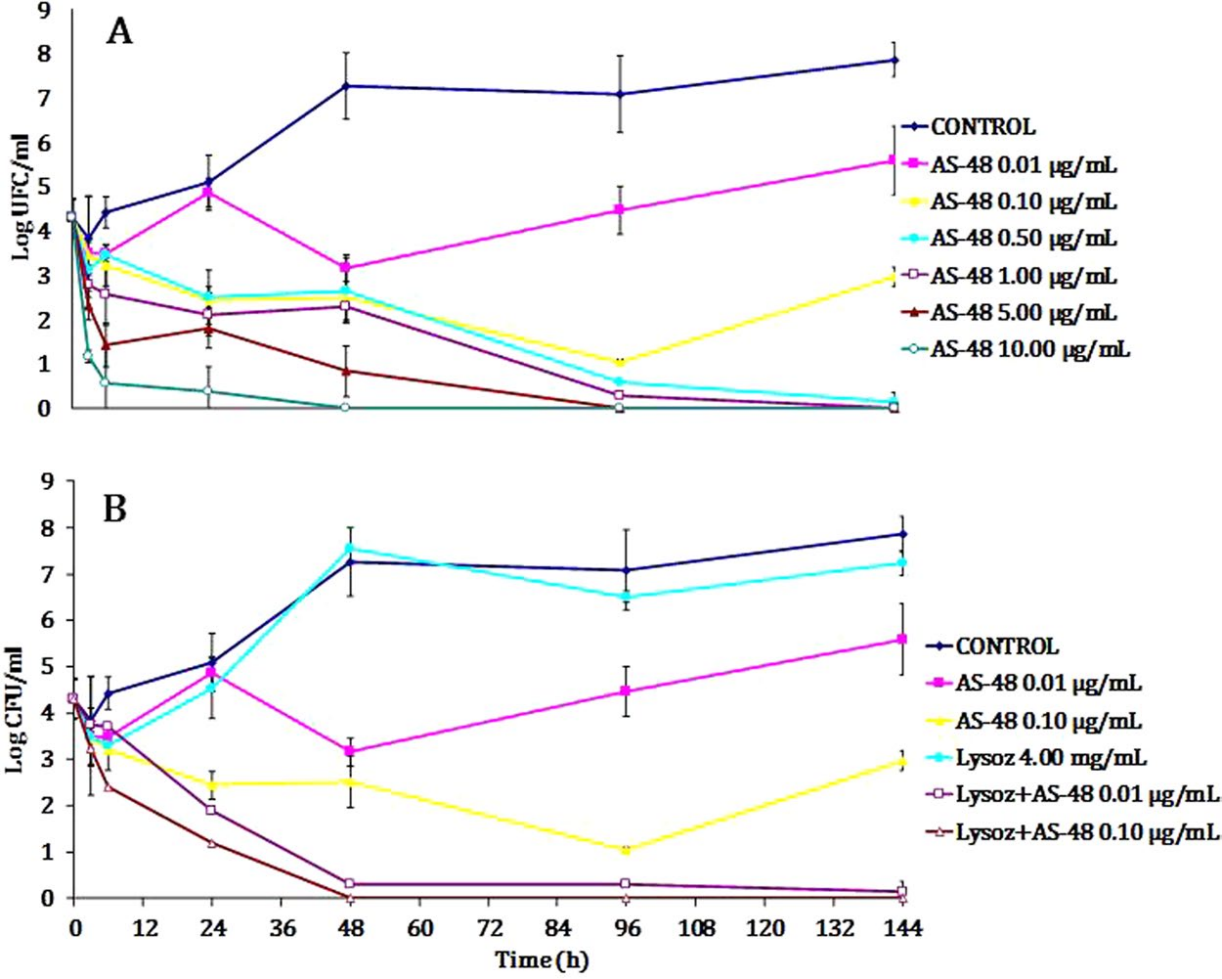
Killing kinetics of AS-48. Growth of *P*. *acnes* (log CFU/mL) after prolongued exposure to different AS-48 concentrations (**A**) or a combination of AS-48 with 4.00 mg/mL lysozyme (**B**). A control with no antimicrobial (dark blue) was used.

*P*. *acnes* P0 was treated with a wide range of AS-48 concentrations (0.01 to 10.00 µg/mL) and the number of viable cells (CFU/mL) measured along time (6 days) (Fig. 5). When AS-48 was used alone, we could determine the absence of viable cells after 48 h incubation when 10 µg/mL were used (Fig. 5A). Smaller concentrations of AS-48 could only achieve this after prolongued incubation time (96 h at 5.00 µg/mL and 144 h at 1.00 µg/mL). When lysozyme (4.00 mg/mL) was included in the test, we could observe the absence of surviving cells after 48 h incubation at only 0.10 µg/mL of AS-48, thus 100-fold diluted (Fig. 5B).

### Cytotoxicity assays

An important concern regarding the warrant use of compounds as therapeuticals is to know their *in vitro* cytotoxicity. For this, two human malignat skin cell lines (melanoma cell line A2058 and fibroblast from normal skin of a patient with a high grade glioma CCD25sk) were incubated with AS-48 at concentrations ranging from 200.00 to 0.048 µg/mL alone and in the presence of lysozyme (4.00 mg/mL) to check their viability by the MTT assay.

**Figure 6:**
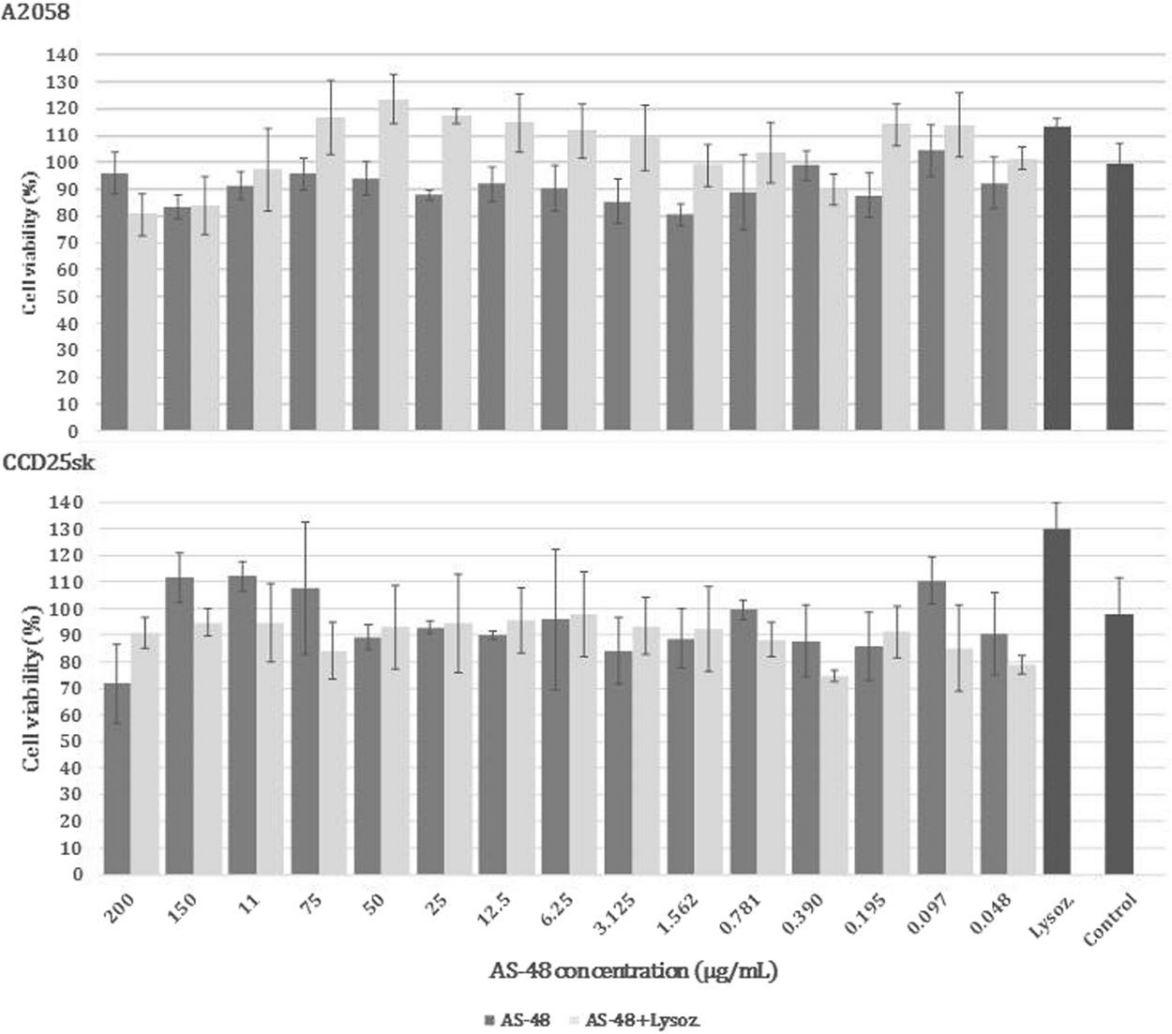
Viability of the A2058 and CCD25sk cell lines (% in relation to control) after the treatment with several concentrations of AS-48 alone or in presence of lysozyme (4.00 mg/mL). Lysoz: control of lysozyme. Control: untreated cells.

Under the experimental conditions used, the two human-skin cell lines were unaffected by the presence of AS-48 at concentrations close to the MIC and showed no signs of viability changes even at the highest concentration tested (200.00 µg/mL) (Fig. 6). Lysozyme alone (4.00 mg/mL) had no effect on these cell lines. According to the statistical analysis no significant differences were observed between treated and control cells (Fig. 6), so we can affirm that AS-48 and lysozyme are not toxic for these cell lines in the condition assayed.

## Discussion

Recent advances on the knowledge of antimicrobial peptides (AMPs) with improved activities are leading these molecules in novel formulations as promising candidates for the treatment of infectious diseases^13,42,43^. Bacteriocins represent one of the most studied microbial defence systems. It is clear from both their abundance and variations that bacteriocins are the weapons of choice in the microbial world for intra- and interspecies antagonism^44^. Their antimicrobial activity is specifically related to its amino acid composition and physical chemical properties, such as positive net charge, flexibility, size, hydrophobicity, and amphipathicity.

With this knowledge in mind, we have explored the susceptibility of several clinical *P*. *acnes* isolates from different sources to the bacteriocin AS-48. The potent activity demonstrated against *P*. *acnes*, encourages us to propose it as a novel and useful candidate for its further pharmacological development against this bacterium. Available experimental data confirm that AS-48 is an amphipathic peptide characterized by a significant proportion of hydrophobic amino acid residues and rich in positively charged residues (8 lysines and 2 arginines), which selectively binds to fluid membranes with a large proportions of anionic phospholipids, as the bacterial membranes, while it leaves intact the rest of normal eukaryotic cell membranes, in which uncharged lipids predominate at the host cell surface^26,45^, being some trypanosomides (*Leishmania* and *Trypanosoma brucei*) the exception^28,46^. Moreover, the AS-48 selectivity for the cell membrane leads to a permeabilization that allows rapid movement of small molecules. This phenomenon difficults the development of resistances and underlines its potential therapeutic value as antibacterial agent, showing simultaneous antibacterial and biofilm-disrupting activities, similarly to those described for other lysine-based molecules^47^.

In a recent study we have described the genotypic and phenotypic profile of several clinical isolates of *P*. *acnes* from different sources, which were grouped into two clusters by Random Amplification of Polymorphic DNA, in correspondence with the phylogroups I and II previously established^33,48^. Remarkably, all the isolates from acne and the majority from opportunistic infections belonged to biotype I-B3 in correlation with the phylotype IA1, which is involved in the pathophysiology of *P*. *acnes* according to the multiplex touchdown analysis performed. No clear relation was observed between the antibiotic resistance of these strains and their subgroup profiles. Indeed, the P3, P11 and P12 strains from acne patiens previously treated with antibiotics, were resistant to erythromycin, while P11 and P12 showed combined resistance to clindamycin^33^. The remarkable susceptibility of these strains to AS-48 in liquid medium (Table 1), in the range of the most sensitive Gram-positive bacteria assayed, suggests that this peptide could be used to effectively inhibit clinical propionibacteria at wery low AS-48 concentrations (below 2.50 µg/mL). In close connection with this matter, we were interested in testing if AS-48 works better in combination with lysozyme. The use of this compound as an antimicrobial peptide enhancer is attracting attention because this combination increases the effectiveness, the spectrum of activity and favors the absence of resistance against them^49–52^. The clear potentiating effect of lysozyme causes a reduction in the MIC of AS-48 against nearly all the strains tested, therefore indicating the suitability of combined therapy.

Recent studies have demonstrated the ability of *P*. *acnes* to form biofilms in different biological media or prosthetic implants, playing an important role in the chronic course of the infections^6,53^. A biofilm is an adherent sessile community of cells attached to a substratum, interface, or each other, embedded in a self-produced polymeric matrix that exhibits an altered phenotype with regard to growth, gene expression, and protein production compared to planktonic cells^35^. Likewise, antimicrobial susceptibility is usually reduced in biofilms, where the cells are much more tolerant to antimicrobial agents than their planktonic counterparts^54,55^. Failures to treat and eradicate bacterial infections are usually due to their biofilm formation capacity^37^. Surprisingly, low concentrations of AS-48 seem to induce the development of a network visible in SEM. Something similar has been described with some antibiotics^56^. Interestingly this net reminds the self-assembled peptide nanonets produced by the human alpha-defensin 6^57^, but here its nature and function remain to be elucidated. The existence of such structures may be in accordance with the biofilm induced *in vitro* by sub-MIC concentrations of a variety of antibiotics in some bacteria, as a global cell response to the stress, and as an inducible resistance mechanism^58^. We have observed that the deleterious effect of AS-48 against *P*. *acnes* is not immediate, therefore this network may be interpreted as an initial defence mechanism of the cells, although finally they end up dying. In our opinion this is not a significant concern in light of the viability assays performed that confirm the progressive and effective death (although not lysis) of the cells, even those growing in a biofilm. Collectively, our data confirm that the mechanism of action is based on membrane binding and permeabilization. The results highlight the AS-48 potential as an antimicrobial agent for the control of dermatological infectious diseases such as acne vulgaris, as its has been demostrated using a range of microscopy and bioassay techniques.

Furthermore, our results confirm that the combination of AS-48 with lysozyme potentiates the bactericidal effect on *P*. *acnes*, with reductions in the AS-48 MICs in 19/20 (95%) cases and a faster killing kinetics at a shorter time (Table 1, Fig. 5). We show a drastic increase of the antimicrobial activity against *P*. *acnes* associated to the increase of cell membrane damage, a mechanism that prevents the rapid development of resistance. More importantly, this combination is also effective on cells growing in a biofilm, with a significant biomass reduction, suggesting its capacity to inhibit the matrix formation and even to disorganize it once formed. Thus, the synergy between these compounds highlights the importance of the cocktail therapies also against biofilms^59^ and shows the advantages of combining molecules with different targets. This further reduces the chances of cytotoxicity in mammalian cells and the development of resistances, in this case by pathogens that colonize and infect the skin^15,25,26,60^. These results are noticeable due to the ability of these persistent bacteria adhered at the implanted medical devices forming a biofilm and causing chronic infection of difficult treatment. The suitability of these compounds as pharmaceuticals has been also explored through determinations of cytotoxicity. This is an essential step to warrant a safe use, and for this we have assayed AS-48, both sole and combined with lysozyme, against human malignant skin cells, which were unaffected not even by high concentrations of AS-48 and lysozyme far above the MIC.

## Conclusions

The results presented in this work are relevant given the increasing impact of antibacterial resistances, providing the basis for developing improved topical formulations to win the game to the acne (an infection with a strong social impact) or to be applied in biofilm-associated infections linked to implants, which are very difficult to prevent and/or treat. Our laboratory results are promising and point at AS-48 as a useful therapeutic agent against this bacterium. Besides, AS-48 combined with lysozyme promotes the cooperative outcomes here described. Further validation of the usefulness of AS-48 in biomedical fields would require *in vivo* studies to evaluate its efficacy, although current findings support the viabilty of incorporating this bacteriocin into a variety of skin disease therapies. It remains to emphasize that AS-48 head-to-tail union confers a great stability and improves its bioactivity. These are valuable characteristics for its future biotechnological applications even in the skin, where large amounts of Na^+^ are stored^61^. Thus, we agree with other authors^43,44^ on the fact that now it is the time for the antimicrobial peptides, and AS-48, alone and preferably in combination with lysozyme, offers an immense medical potential, even against pathogens resistant to conventional antibiotics.

## Methods

### Bacterial strains

The clinical isolates of *P*. *acnes* used in this work, isolated from different patients of two hospitals in southern Spain, have been previously genotyped^33^. These strains were isolated from inflammatory acne, wound exudates, opportunistic infections or as contaminants (Table 1). *P*. *acnes* P0 was obtained from the Spanish Collection of Type Cultures (CECT 5684/ATCC 6919). The study protocol was carried out in accordance with the Declaration of Helsinki. This was a non-interventional study with no additional investigation to routine procedures. Biological material was only used for standard diagnostics following physicians’ prescriptions. No additional sampling or modification of the routine sampling protocol was performed. Data analyses were carried out using an anonymous database.

All bacterial strains were cultured at 37 °C in Differential Reinforced Clostridial Medium (DRCM) (Scharlau), Brain Heart Infusion (BHI, Scharlau) or Wilkins-Chalgren anaerobe broth (WC) (Oxoid). Anaerobic conditions were established using AnaeroPack system sachets (Thermo Scientific).

### Bacteriocin AS-48 purification

AS-48 was purified from cultures of the enterococcal UGRA10 strain^62^ on Esprion 300 plus glucose (1%) (DMV Int., Veghel, Netherland) in a pH-controlled device in the conditions previously established^63^. Briefly, the fermentation supernatant was purified by cationic exchange chromatography on a Carboxy Methyl Sepharose matrix (CM25, GE Amershan) and desalted and concentrated using reversed-phase chromatography on C18 silica beads (Water). The bacteriocin was purified to homogeneity by RP-HPLC^64^. This protocol typically yields around of 100 mg/L AS-48^63^. The protein concentration of the purified AS-48 samples was determined by measuring UV absorption at 280 nm in a Nanodrop 2000 (Thermo Scientific).

### Biological activity assays

The susceptibility range of the isolates to AS-48 and to AS-48 plus lysozyme has been investigated. The minimal inhibitory concentration (MIC) was determined using the broth microdilution method according to the Clinical and Laboratory Standard Institute (CLSI)^65^ guidelines for anaerobic bacteria, although using DRCM to favor its growth. For this, half-decreasing concentrations of purified AS-48 samples (10.00 to 0.018 µg/mL) alone or in combination with lysozyme (0.40 mg/mL) (Sigma) were assayed in 96-well microtiter plates using an initial inoculum of the indicator strains of 5 × 10^5^ CFU/mL. Microtiter trays were incubated at 37 °C for 72 h under anerobic conditions. After incubation, any well showing turbidity measured in a Tecan Spectrophotometer (Sunrise) was considered to exhibit bacterial growth. In both cases, three or more independent experiments were performed starting from different protein stocks.

### Biofilm composition

Biofilm-stability assays were performed on 96-well conical-bottoned microtitre plates under anaerobic conditions for 48 h in WC liquid medium (100 µl), inoculated with several *P*. *acnes* isolates. Once adhered, the medium was replaced by fresh medium and supplemented with different dispersal agents^36^: 200 mM sodium metaperiodate (Alfa Aesar, Ward Hill, MA, USA), 250 µg/mLprotease K (Acros Organics) or 250 µg/mL RNase-free DNase I (Takara Bio Inc., Shiga, Japan) and re-incubated for 2 h at 37 °C. AS-48 at 20.00 µg/mL was used too as a dispersant agent. After the treatments, the wells were visualized. If some dispersal agent disorganized the biofilms, the cells appeared as a button at the bottom. The medium without supplement served as a negative control. Each experiment was performed twice independently for each strain.

### Biofilm formation examined by scanning electron micrograph (SEM and TEM)

*P*. *acnes* was inoculated (5%) into series of WC tubes (3 mL), containing or not a small glass slide (10 × 10 mm). Three different AS-48 concentrations (0.10, 1.00, and 10.00 µg/mL) were added at the beginning of the growth (T_0_) or after 24 h (T_24_) in anaerobiosis and then re-incubated.

For SEM, the slides were fixed with glutaraldehyde in PBS (3%) at pH 7.2 at 4 °C overnight and then, washed three times and suspended in PBS for fixation with osmium tetroxide (1%) for 1 h at room temperature and gradually dehydrated in increasing concentrations of ethanol. After this, biological samples were dried by the method for critical point^66^ with carbon dioxide in a dessicator Polaron 7501 CPD and the samples were coated by evaporation on an evaporator coal Coal EMITECH K975X and viewed and imaged with high-resolution SEM (FESEM Zeiss Supra 40VP) equipped with an EDX AZTEC microanalyser.

For observation in TEM, the cells treated with different amounts of purified AS-48, were pre-fixed with a 2.5% (v/v) glutaraldehyde solution (Merck, Madrid, Spain) in 0.1 M sodium cacodylate buffer pH 7.2 (Merck) at 4 °C for 2 h, followed by three washes in the same cacodylate buffer. Fixed samples were prepared for electron microscopy examination at the Scientific Instruments Centre of the University of Granada. Samples were also fixed with 1% with OsO_4_ in the same buffer at 20 °C for 3 h, dehydrated, and embedded into EMBed 812 R resin (Electron Microscopy Science, Hartfield, PA). Resin blocks were sectioned and mounted on copper grids contrasted with 1% uranyl acetate (Merck) and stained with lead citrate (Merck). Finally, they were viewed under a Carl Zeiss 902 transmission electron microscope (Carl Zeiss, Jena, Germany) operating at 80 kV.

### Cell-viability assays by flow cytometry

To determine the effect of AS-48 on the membrane of biofilm forming cells, vital stain with a solution of 100.00 μg/mL of orange acridine plus propidium iodide (1:1) in PBS was used. Propidium iodide is not permeant to live cells, it can only penetrate cells with their membrane damaged and subsequently binds DNA producing fluorescence upon excitation with UV light at 535 nm. 48 h cultures grown in anaerobiosis in 2 mL of WC medium inoculated (5%) with *P*. *acnes* were performed in 10 mL glass tubes to allow the formation of adherent cells attached to each other, embedded in a self-produced hydrated polymeric matrix. Then, the supernantants were removed and fresh medium with half-decreasing concentrations of AS-48 samples (16.00 to 0.065 µg/mL) alone or with lysozyme (4.00 mg/mL) was added to the cultures and re-incubated for 24 h at 37 °C. Then, the supernatant was removed and the cells washed with PBS twice, and stained with a solution of 100.00 μg/mL orange acridine plus propidium iodide (1:1) in PBS in a 1:25 ratio^41^. The viability of the cells in the biofilm after resuspension was evaluated by fluorescence-activated cell sorting (FACS) flow cytometry. Titration and staining tests were performed on a Becton Dickinson FACS Calibur flow cytometer (BD Bioscience, San Jose, CA, USA) equipped with a 488 nm argon laser and with two light-scatter detectors that measured forward (FSC) and side scatter (SSC). Fluorescence was detected by PMT detectors with appropriate fluorescence filter sets for FL-1 (530/30 BP for orange acridine) and for FL-3 (661/16 BP for propidium iodide). The threshold was set on SSC. Data were stored as list mode files and analysed off-line using the CellQuest software (Becton Dickinson).

### Killing kinetics of AS-48

*P*. *acnes* P0 (CECT 5684) was grown under anaerobic conditions at 37 °C until the cell culture reached approximately 10^5^ CFU/mL. At this point, the culture was splitted and treated with different AS-48 concetrations (0.01, 0.10, 0.50, 1.00, 5.00 and 10.00 µg/mL). Additionally, in tubes containing 0.01 and 0.10 µg/mL of AS-48, a final concentration of 4.00 mg/mL lysozyme was added. Samples were taken at different time points and plated in triplicate on BHI. The number of colonies (CFU/ml) was counted after incubation of the plates for 48 h at 37 °C under anaerobic conditions.

### Determination of cytotoxicity (MTT assay)

The *in vitro* effect of different AS-48 concentrations (200, 150, 100, 75, 50, 25, 12.50, 6.25, 3.12, 1.56, 0.78, 0.39, 0.195, 0.098 and 0.049 µg/mL) alone or in combination with lysozyme (4.00 mg/mL) on two skin eukaryotic cell lines, A2058 (ATCC® CRL-11147™) and CCD25sk (ATCC® CRL-1474™), was performed by the Cytotoxicity Service of the Fundación Medina (Granada, Spain). The viability of the cell lines, was evaluated using the colorimetric MTT assay (Sigma, Aldrich), measuring the activity of cellular enzymes that reduce the tetrazolium dye to its insoluble form formazan, giving a purple color.

Cells seeded in 96-well plate at a density of 1 × 10^4^ cells/well in 200 μL of appropriate culture medium were incubated overnight at 37 °C in 5% CO_2_. Medium was replaced and cells exposed to medium containing 2.40 μL of different concentrations of AS-48 added or not of lysozyme, to be assayed for 24 h. As positive control, 8 mM methyl methanesulfonate (MMS) was used and 0.5% DMSO as negative control. A control with lysozyme (4.00 mg/mL) alone was also carried out. As standards (internal control) a doxorubicin curve was used. Each result was calculated from three independent measurements. When compounds and controls were added, plates were incubated at 37 °C in 5% CO_2_ incubator for 72 h. After each incubation time, the medium was removed from wells and MTT solution was added. Cells were incubated with MTT for 3 h. The formazan crystals created after incubation were dissolved in DMSO (dimethyl sulfoxide). Absorbances of obtained colored solutions were measured under 570 nm wavelength using a multireader Victor TM. The results of viability are displayed in percentage compared to control (100%). The results are presented as the means ± standard deviations.

### Statistical analysis

The experimental results carried out at least in three independent tests, were subjected to statistical analysis using the IBM SPSS statistics 20 (IBM, Spain). Data relative to the antimicrobial activity of AS-48 alone or in combination with lysozyme were subjected to ANOVA, using AS-48 or AS-48 plus lysozyme as factor. Tukey was used as a post-hoc test to determine significant differences between the control and the treatments. The criterion *p* < 0.05 was used to determine the statistical significance.
